# Influences of Drying Methods on Water‐Soluble, Insoluble, and Total Dietary Fiber Contents and Physicochemical Properties of Mung Bean (*Vigna radiata*), Guava (*Psidium guajava*), and Bitter Gourd (*Momordica charantia*)

**DOI:** 10.1155/ijfo/3344999

**Published:** 2026-04-18

**Authors:** Eranga Gunawardhana, Rumesh Liyanage, S. B. Navaratne, Lasanthi Jayathunge, Nilanthi Wijewardane, Shakila Dilshani Rukunayaka, Imalsha Dilshani Weerasinghe

**Affiliations:** ^1^ Department of Biosystems Technology, University of Sri Jayewardenepura, Homagama, Western Province, Sri Lanka, sjp.ac.lk; ^2^ Department of Food Science and Technology, University of Sri Jayewardenepura, Nugegoda, Western Province, Sri Lanka, sjp.ac.lk; ^3^ Research and Development Centre, National Institute of Post-Harvest Management, Anuradhapura, North Central Province, Sri Lanka, ncbir.pl

**Keywords:** dietary fiber, heat pump drying, hot-air drying, plant food sources, proximate composition, sun drying

## Abstract

The mung bean (*Vigna radiata*), guava (*Psidium guajava*), and bitter gourd (*Momordica charantia*) are notable legumes, fruits, and vegetables that are widely cultivated in Sri Lanka and are well‐known for having high levels of dietary fiber. They have a high tendency to utilize their dried powders or extracted fiber concentrates to develop dietary fiber–incorporated functional foods to enhance the consumption of dietary fiber. Thus, in this study, plant sources underwent drying using three frequently practiced drying methods in Sri Lanka, hot‐air drying, heat pump drying, and sun drying, to investigate the impacts of the application of drying methods on dietary fiber composition, along with the proximate composition, functional properties, and color characteristics of dried samples. The results revealed that the hot‐air drying preserved the highest soluble dietary fiber content in all plant sources (mung bean: 4.25%, guava: 8.12%, and bitter gourd: 10.95%). Sun drying imparted the highest insoluble dietary fiber contents, particularly in guava (38.65%) and bitter gourd (51.04%), whereas bitter gourd showed the highest total dietary fiber (56.65%) with sun drying. Mung bean had a superior crude protein content of 32.57% under heat pump drying but was least effective in retaining guava protein (4.19%). The crude protein content varies considerably across the plant sources and drying methods, while the crude fat and ash contents show no significant variations across the drying methods. Heat pump drying maintained superior water‐holding capacities, oil‐holding capacities, and swelling water capacities in all plant sources, enhancing their functional properties. Color analysis of the dried plant sources revealed that hot‐air‐dried samples had the darkest coloration, whereas heat pump drying maintained the best natural hues of the dried materials. Our findings provide valuable insights for optimizing drying methods to enhance the important nutritional, functional, and visual qualities of dietary fiber–rich plant sources.

## 1. Introduction

The nutritionist E. H. Hipsley first coined the term “dietary fiber” (DF) in 1953 to describe the consumption of indigestible elements of plant cell walls [1]. DF mainly comprises various plant‐based components, including nonstarch polysaccharides like cellulose and hemicellulose and noncellulosic polymers such as pectin, gums, mucilage materials, and lignin [2, 3]. According to the solubility in water, two primary fractions in DF exist as insoluble dietary fiber (IDF), which is usually found in higher quantities in plant sources, and soluble dietary fiber (SDF), which amounts to small proportions [4]. Human or animal gastrointestinal tract enzymes resist digesting DFs since these plant components used as food remain undigestible. However, they undergo partial or complete fermentation by the intestinal bacteria in the large intestine [5, 6]. Therefore, DF is generally referred to as “roughage materials” or “resistant polysaccharides” due to its indigestibility in the human body [2, 7]. Beyond their traditional perception as roughage materials, DF is now recognized as an essential functional ingredient that delivers significant health benefits and improves food properties. This rising awareness of the vital functionality of DF for food improvement alongside health benefits shapes a new acceptance trend for the plant sources rich in DFs.

In the past few decades, extensive studies have demonstrated that DF acts as a central element for human health promotion [8–10]. Particularly, health benefits associated with DF enable diabetes management, obesity treatment, gut disorder management, and alleviating various cancer types [6]. Moreover, DF‐rich diets reduce body cholesterol and glycemic levels, shortening digestion time, promote beneficial intestinal microflora, and capture harmful substances to perform as a protective system that absorbs mutagenic and carcinogenic substances [10]. Additionally, DF application into food formulations enhances and modifies the food properties while delivering functionalities such as water‐holding capacity (WHC), swelling water capacity (SWC), oil‐holding capacity (OHC), emulsification, viscosity, texture, and gel formation and enhances overall quality [11].

The history of agriculture in Sri Lanka extends more than 2500 years, and it is thought that this agricultural foundation is where people’s livelihoods initially began [12], and the country’s economy primarily depends on agricultural practices [13, 14]. As a result, many different DF‐rich crop varieties are commercially grown across the country. Among these plant sources, mung bean (*Vigna radiata*), guava (*Psidium guajava*), and bitter gourd (*Momordica charantia*) are well‐known sources for their nutritional value, particularly for their high DF contents [15–18]. They exhibit significant health‐promoting properties such as antidiabetic, antimetabolic disorder, antiviral, antimicrobial, anticancer, and antiobesity properties [17, 19].

The vitality of this study is its focus on quantifying the SDF, IDF, and TDF content of plant sources and examining the impacts of hot‐air drying (HAD), heat pump drying (HPD), and sun drying (SD) on the SDF, IDF, and TDF content in mung bean, guava, and bitter gourd. Among the various processing practices, drying is the most commonly utilized as a preservation technique to preserve plant materials as a dried product or powder, which can be later used as key ingredients in food formulations, such as dairy products, breakfast cereals, and dietetic food formulations [20, 21]. Usually, the preservation takes place by the removal of moisture from plant sources [22]. Various types of drying techniques have been studied by many researchers to be applied to preserve and improve the products’ qualities, especially in DF‐rich plant materials [20, 21]. It is a challenge to maintain nutritional values, particularly in fruits and vegetables such as guava and bitter gourd, by using drying techniques [20].

On the one hand, HAD, also known as convective drying, is a widely used oven drying method for plant materials due to its simplicity in design, low operational cost, broad range of applicability, and ease of operation [23]. On the other hand, HPD is also used in the food industry as a useful and promising drying method to preserve heat‐sensitive plant materials by providing the optimum conditions, due to its capability of operating over a wide range of temperatures and humidities, which makes HPD a high‐energy and highly efficient drying technique that offers less quality loss of dried products [24]. In addition to the mechanical drying techniques, SD is a popular low‐cost drying method that has been intensively utilized in tropical countries since ancient times. However, some qualities of SD plant materials, such as color, texture, and drying duration, may result in differences between them because they rely on ambient environmental conditions, which naturally fluctuate [20].

Given the complex interactions among soluble, insoluble, and total DFs and physicochemical properties in the selected DF‐rich plant sources of mung bean (*Vigna radiata*), guava (*Psidium guajava*), and bitter gourd (*Momordica charantia*), applying commonly used drying methods of HAD, HPD, and SD is expected to enhance the selection of an appropriate drying method that affects the retention of DF and nutritional compounds, which are crucial to preserving the health benefits linked with these plant‐based sources. Therefore, this study aims to identify the appropriate drying method for each selected plant source by evaluating water‐soluble, insoluble, and total DF contents, as well as the primary physicochemical properties such as WHC, OHC, water‐swelling capacity, and color variations after drying. These values will be used to determine how effectively each drying method retains the functional and nutritional properties of DF‐rich plant materials.

## 2. Materials and Methods

### 2.1. Plant Sources Collection and Preparation

The study was conducted at the Faculty of Technology, University of Sri Jayewardenepura (USJ), Sri Lanka, in collaboration with the Research and Development Centre, National Institute of Post‐Harvest Management (NIPHM), Sri Lanka. The mung bean (*Vigna radiata*), guava (*Psidium guajava*), and bitter gourd (*Momordica charantia*) were subjected to this study, which were from selected cultivation sites located in Anuradhapura District, North Central Province, Sri Lanka. The storage of mung beans occurred at ambient temperature, whereas guava and bitter gourd were kept at 4°C for no more than 6 h before sample preparation. After removing the seeds, the guava and bitter gourd were washed thoroughly with portable water and manually cut into thin slices with a thickness of approximately 3‐4 mm using a stainless‐steel knife. To maintain the thickness of the slices as uniform as possible, a premeasured stainless‐steel spacer was used as a guide throughout the cutting process to ensure consistency among all samples.

### 2.2. Drying Experiments

**FIGURE 1:**
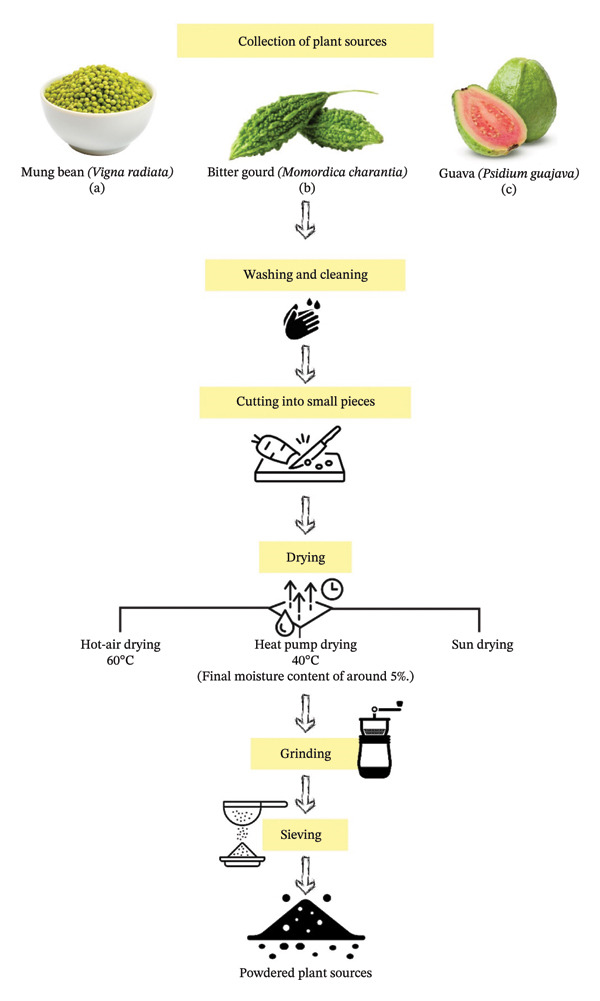
The illustration represents the sequence of sample preparation steps before applying the hot‐air oven, heat pump, and sun drying methods. (a) Mung beans, (b) bitter gourds, and (c) guava fruits are at their maturity levels. All samples were rinsed with potable water, deseeded, and cut with a stainless‐steel knife before drying.

The plant sources were subjected to three distinct drying methods to evaluate the variations in DF contents and physicochemical properties of their dried powders. These drying methods included two oven drying methods, HAD and HPD, alongside the conventional drying method of SD, which was applied to DF‐rich plant sources. A flow diagram of the preparation of plant sources by employing drying methods is presented in Figure 1.

#### 2.2.1. HAD

Approximately 1000 g of cleaned and sliced samples were dried in a hot‐air oven (Memmert, Lording‐Model 30–60, USA) at 60 ± 2°C [25, 26]. The samples were evenly distributed on stainless‐steel drying trays, with each tray dedicated to a specific plant source. To maintain uniformity, all trays were placed in identical locations in the oven, ensuring consistent drying conditions across the plant sources. The initial moisture contents of fresh plant materials are also listed in Table 1 (AOAC 925.45). Drying was continued until the samples reached a final moisture content of around 5%, as confirmed using a moisture analyzer (Ohaus, MB25, USA).

**TABLE 1:** Initial moisture contents of mung bean, guava, and bitter gourd.

Source	Initial moisture content (%)
Mung bean (*Vigna radiata*)	14.25 ± 0.42
Guava (*Psidium guajava*)	89.32 ± 0.50
Bitter gourd (*Momordica charantia*)	92.19 ± 0.13

*Note:* Means ± standard deviation (*n* = 5). All results were reported on a dry basis (db).

#### 2.2.2. HPD

Approximately 1000 g of cleaned and sliced samples were dried in a heat pump dryer (MY‐03RD‐16L, China) at 40 ± 2°C (one batch at a time) [27]. The samples were evenly distributed on stainless wire trays, with each tray placed on the same rack in the dryer to maintain uniform drying conditions across all plant sources. The initial moisture contents of fresh plant materials are also listed in Table 1 (AOAC 925.45). Drying was continued until the samples reached a final moisture content of around 5%, as confirmed using a moisture analyzer (Ohaus, MB25, USA).

#### 2.2.3. SD

Approximately 1000 g of cleaned and sliced samples were separately sun‐dried for 3 to 5 days between March and April, a period characterized by the highest daily sunshine intensity and more than 7 h of clear skies in the dry zone of Sri Lanka [28]. The samples were evenly spread on a mat positioned in an elevated, open area on the laboratory premises of the National Institute of Post‐Harvest Management, Anuradhapura (the area belongs to the Low Country Dry Zone, Agro‐Ecological Region: DL1b), ensuring uninterrupted exposure to direct sunlight, with ambient temperatures ranging between 27°C and 33°C and relative humidity ranging between 50% and 65%. The initial moisture contents of fresh plant materials are also listed in Table 1 (AOAC 925.45). Drying was continued until the samples reached a final moisture content of around 5%, as confirmed using a moisture analyzer (Ohaus, MB25, USA).

### 2.3. DF Composition

The SDF, IDF, and TDF contents of dried plant sources were analyzed enzymatically–gravimetrically (AOAC 991.43) using the “Megazyme K‐TDFR‐200A total dietary fiber assay kit.”

#### 2.3.1. Determination of IDF

Duplicates of precisely weighed samples (1.000 ± 0.005 g) were subjected to sequential hydrolysis with heat‐stable α‐amylase (at 98°C–100°C for 30 min), protease (at 60°C for 30 min), and amyloglucosidase (at 60°C for 30 min). IDF residue was separated by vacuum filtration through glass crucibles (porosity No. 2), while the filtrate was preserved for the subsequent analysis of SDF. The residue‐containing crucibles were gently rinsed with 10‐mL portions of 95% ethanol and acetone and dried at 105°C to a constant weight. Then, residual protein and ash contents were estimated using the AOAC 2001.11 and AOAC 942.05 methods, respectively. The following formula was subsequently used to calculate the IDF content (%) of dried plant sources [29].

(1) IDF %=r12+r/2−P−A−B/m12+m2×100,

where *r*1 is the residue weight 1 from Sample 1 (in grams), *r*2 is the residue weight 2 from Sample 2 (in grams), m1 is the sample weight 1 (in grams), *m*2 is the sample weight 2 (in grams), *A* is the ash weight from Residue 1 (in grams), *P* is the protein weight from Residue 2 (in grams), and *B* is the blank weight (in grams).

#### 2.3.2. Determination of SDF

The filtrates retained from the IDF separation were subjected to precipitate SDF by mixing with the preheated 95% ethanol (at 60°C). The resulting precipitates were filtered through a glass crucible (porosity No. 2), followed by sequential rinsing with 15 mL portions of 78% ethanol, 95% ethanol, and acetone, and residue‐containing crucibles were dried at 105°C to a constant weight. Then, residual protein and ash contents were estimated using the AOAC 2001.11 and AOAC 942.05 methods, respectively. The following formula was subsequently used to calculate the SDF content (%) of dried plant sources [29]:

(2) SDF %=r12+r/2−P−A−B/m12+m2×100,

where *r*1 is the residue weight 1 from Sample 1 (in grams), *r*2 is the residue weight 2 from Sample 2 (in grams), *m*1 is the sample weight 1 (in grams), *m*2 is the sample weight 2 (in grams), *A* is the ash weight from Residue 1 (in grams), *P* is the protein weight from Residue 2 (in grams), and *B* is the blank weight (in grams).

#### 2.3.3. Determination of Total Dietary Fiber (TDF)

Duplicates of precisely weighed samples (1.000 ± 0.005 g) were subjected to sequential hydrolysis with the digestive enzyme by following the same procedure applied for the IDF determination. Then, the samples were mixed with four volumes of preheated 95% ethanol (at 60°C) to precipitate the TDF. The resulting precipitates were filtered through a glass crucible (porosity No. 2), rinsed with 15‐mL portions of 78% ethanol, 95% ethanol, and acetone, and dried at 105°C to a constant weight. Then, residual protein and ash contents were estimated using the AOAC 2001.11 and AOAC 942.05 methods, respectively. The following formula was subsequently used to calculate the SDF content (%) of dried plant sources [29]:

(3) TDF %=r12+r/2−P−A−B/m12+m2×100,

where *r*1 is the residue weight 1 from Sample 1 (in grams), *r*2 is the residue weight 2 from Sample 2 (in grams), *m*1 is the sample weight 1 (in grams), *m*2 is the sample weight 2 (in grams), *A* is the ash weight from Residue 1 (in grams), *P* is the protein weight from Residue 2 (in grams), and *B* is the blank weight (in grams).

### 2.4. Proximate Composition

The proximate composition of dried plant sources, including crude protein, crude fat, and ash contents, was analyzed gravimetrically by following the recommended AOAC methods.

#### 2.4.1. Determination of Crude Protein

The crude protein of dried plant sources was estimated using the Kjeldahl method (AOAC 2001.11) by digesting approximately 2 g of the sample in concentrated sulfuric acid at 350°C for 4 h with the aid of a catalyst. The digested sample was then distilled off using a semimicro Kjeldahl distillation apparatus, and the collected distillate was titrated against standard hydrochloric acid. The following formulas were subsequently used to calculate the crude protein content (%) [29]:

(4) nitrogen %=V12−V×n11×F×MWnWs×10,

where *V*1 is the volume of the sample titrate (in milliliters), *V*2 is the volume of the blank titrate (in milliliters), *n*1 is the normality of hydrochloric acid, *F*1 is the acid fraction, *M* *W* *n* is the molecular weight of nitrogen, and *W* *s* is the sample weight (in grams).

(5) Crude protein %=N×6.2510×,

where *N* is the nitrogen content of the sample (as a percentage).

#### 2.4.2. Determination of Crude Fat

The AOAC 920.39 method was employed to determine the crude fat of dried plant sources. Approximately 5 g of sample was filled into a thimble and placed in the Soxhlet extractor to extract fat into a round‐bottom flask by refluxing it with petroleum ether for 5 h. Once the refluxing was done, the round‐bottom flask containing the fat was dried at 105°C to a constant weight. Then, the crude fat content was expressed as a percentage (%) using the formula outlined as follows [29]:

(6) Crude fat %=W12−WWs×100,

where *W*1 is the weight of the round‐bottom flask with the contents (in grams), *W*2 is the weight of the round‐bottom flask (in grams), and *W* *s* is the sample weight (in grams).

#### 2.4.3. Determination of Ash

The ash content of dried plant sources was determined using the AOAC 942.05 method, by incinerating approximately 2 g of sample in a muffle furnace (Hobersal, HD‐230, Spain) at 525°C for 5 h. The resulting ash content was then obtained as a percentage using the formula as follows [29]:

(7) ash %=W12−WWs×100,

where *W*1 is the weight of the crucible with the contents (in grams), *W*2 is the weight of the crucible (in grams), and *W* *s* is the sample weight (in grams).

### 2.5. Determination of WHC

The method used by de Moraes Crizel et al. [30] was used as the basis for this study to determine the WHC of dried plant sources after performing some modifications. Approximately 0.5 g of the sample was placed in a centrifuge tube, 15 mL of distilled water was added, and the sample was left at room temperature for 24 h. After hydration, the sample was centrifuged at 3000 g for 20 min, and the supernatant was carefully removed to obtain the remaining residue weight. The WHC was calculated as g g^−1^ using the equation as follows:

(8) WHC=Wr−WsWd ,

where WHC is the water‐holding capacity (grams per gram), *W* *r* is the residue weight (in grams), *W* *s* is the initial weight of the sample (in grams), and *W* *d* is the dried weight of the sample (in grams).

### 2.6. Determination of OHC

The method outlined by de Moraes Crizel et al. [30] was used as the basis for this study to determine the OHC of dried plant sources with some modifications. Approximately 0.5 g of sample was placed in a centrifuge tube, 15 mL of sunflower oil was added, and it was left at room temperature for 24 h. The sample was centrifuged at 3000 g for 20 min, and the supernatant was then carefully removed to measure the remaining residue weight. The OHC was obtained as g g^−1^ using the equation as follows:

(9) OHC=Wr−WsWd ,

where OHC is the oil‐holding capacity (in grams per gram), *W* *r* is the residue weight (in grams), *W* *s* is the initial weight of the sample (in grams), and *W* *d* is the dried weight of the sample (in grams).

### 2.7. Determination of SWC

The method used by Jongaroontaprangsee et al. [25] was used as the basis for this study to determine the SWC of dry plant sources with some modifications. Approximately 0.05 g of the sample was placed inside a graduated cylinder and 5 mL of distilled water. The sample was left undisturbed at room temperature for 18 h before measuring its volume when equilibrium was reached. The SWC was expressed as mL g^−1^ and calculated using the formula as follows:

(10) SWC=VsWd,

where SWC is the swelling water capacity (in milliliters per gram), *V* *s* is the volume occupied by the sample (in milliliters), and *W* *d* is the dried weight of the sample (in grams).

### 2.8. Color Measurement

A digital colorimeter (Konica Minolta Sensing, Inc., CR‐400, Japan) equipped with a D65 standard illuminant and 2° standard observer was used to perform the colorimetric attributes of the plant sources before and after drying. Calibration of the instrument was conducted using a white calibration tile before obtaining measurements. A measurement aperture of 8 mm was used to obtain color parameters and expressed using CIE Lab coordinates. Color attributes (*L* ^∗^, *a* ^∗^, *b* ^∗^) of HAD, HPD, and SD samples were measured in five replicates, and the mean values of *L* ^∗^ (lightness), *a* ^∗^ (red green), and *b* ^∗^ (yellow blue) were recorded [31]. The method used by Dadalı et al. [32] and the method used by Coklar et al. [33] were used for calculating the total color change and the browning index (BI) values, respectively.

### 2.9. Statistical Analysis

Results were expressed as mean ± standard deviation. Parametric data were analyzed using the analysis of variance (ANOVA) at a confidence level of 95%. Means were separated using Tukey’s test with a *p* < 0.05 significance level. All the data were analyzed using Minitab statistical software version 19.

## 3. Results and Discussion

### 3.1. Influences of Drying Methods on Dietary Fiber Composition

Table 2 shows the influences of different drying methods, such as HAD, HPD, and SD on the composition of the SDF, IDF, and TDF of mung bean, guava, and bitter gourd.

**TABLE 2:** Variations of the DF composition of dried mung bean, guava, and bitter gourd from different drying methods.

Source	Drying method	SDF (%)	IDF (%)	TDF (%)
Mung bean (*Vigna radiata*)	HAD	4.25 ± 0.08^aF^	19.42 ± 0.30^aE^	23.66 ± 0.46^aF^
	HPD	4.19 ± 0.03^abF^	15.42 ± 0.12^bF^	19.52 ± 0.07^bG^
	SD	4.09 ± 0.06^bF^	15.77 ± 0.09^bF^	19.85 ± 0.06^bG^

Guava (*Psidium guajava*)	HAD	8.12 ± 0.05^aB^	31.11 ± 0.13^bD^	39.31 ± 0.08^bD^
	HPD	7.18 ± 0.01^bC^	30.73 ± 0.58^bD^	38.09 ± 0.57^cE^
	SD	5.54 ± 0.13^cF^	38.65 ± 0.15^aB^	44.46 ± 0.16^aC^

Bitter gourd (*Momordica charantia*)	HAD	10.95 ± 0.13^aA^	36.17 ± 0.07^cC^	47.42 ± 0.03^aB^
	HPD	6.26 ± 0.04^bD^	38.15 ± 0.05^bB^	44.51 ± 0.11^cC^
	SD	5.62 ± 0.02^cE^	51.04 ± 0.12^aA^	56.65 ± 0.11^aA^

*Note:* Means ± standard deviation (*n* = 3). Results of the two‐way ANOVA‐based drying method. Different letters after mean values in columns represent significant differences at *p* < 0.05. Lowercase letters indicate statistical differences between sources, and capital letters describe the combined effect of both source and drying method. All results were reported on a dry basis (db).

Abbreviations: HAD, hot‐air drying; HPD, heat pump drying; IDF, insoluble dietary fiber; SD, sun drying; SDF, soluble dietary fiber; TDF, total dietary fiber.

#### 3.1.1. The Impact of Drying Methods on the DF Composition of Mung Bean

The results relevant to the mung bean exhibited that the HAD showed the highest SDF content (4.25%), followed by HPD (4.19%) and SD (4.09%). This effect was due to the controlled and elevated temperature conditions of the oven, which enhanced the solubilization of the DF components while reducing degradation [34]. The comparatively reduced SDF concentration in all three SD plant sources illustrates the ineffectiveness of uncontrolled drying conditions, which result in extended exposure to heat and light [35]. As highlighted by Kumar et al. [36], elevated temperatures can indirectly enhance the SDF content by degrading other food components. The observed rise in the SDF concentration in the HAD mung bean seems to be correlated with the decrease in nonfiber components in the HAD mung bean sample, where considerably lower crude protein, fat, and ash contents were observed compared to the other employed drying methods, as tabulated in Table 3. In terms of the IDF and TDF contents of the mung bean, it was shown that the HAD outperformed and contained significantly the highest (*p* < 0.000) IDF (19.42%) and TDF (23.66%) content compared to the other two drying methods, showcasing its superiority in preserving these DF components. A similar trend in the IDF and TDF contents seen in mung bean was mirrored by Ujong et al. [37], who demonstrated a similar pattern in crude fiber content for two date varieties dried using HAD and SD. Their findings reveal that HAD samples have a high fiber content ranging from 5.45% to 8.57%, while SD samples range from 3.57% to 6.78% [37]. Similarly, the experimental work of Ladi et al. [38] found 5.15% of SD tomato flour and 6.37% of HAD tomato flour content, indicating consistent results with this study. This supports the notion by Garcia‐Amezquita et al. [34], which signifies that HAD causes the yields of DF extraction, especially insoluble compounds, to increase using various thermal processes like hot‐air circulation in the oven.

**TABLE 3:** Variations of the crude fat, crude protein, and ash contents of dried mung bean, guava, and bitter gourd from different drying methods.

Source	Drying method	Crude protein (%)	Crude fat (%)	Ash (%)
Mung bean (*Vigna radiata*)	HAD	28.15 ± 0.67^cC^	0.81 ± 0.03^aB^	3.93 ± 0.13^aB^
	HPD	32.57 ± 0.43^aA^	0.83 ± 0.02^aB^	4.10 ± 0.25^aB^
	SD	31.05 ± 0.34^bB^	0.82 ± 0.04^aB^	4.02 ± 0.13^aB^

Guava (*Psidium guajava*)	HAD	5.36 ± 0.29^aE^	0.83 ± 0.02^aB^	3.53 ± 0.13^bB^
	HPD	4.19 ± 0.34^bF^	0.82 ± 0.04^aB^	3.54 ± 0.06^bB^
	SD	5.15 ± 0.31^aE^	0.83 ± 0.03^aB^	3.72 ± 0.10^aB^

Bitter gourd (*Momordica charantia*)	HAD	12.03 ± 0.36^aD^	1.36 ± 0.26^aA^	9.66 ± 1.59^aA^
	HPD	11.74 ± 0.49^aD^	1.37 ± 0.09^aA^	9.74 ± 0.13^aA^
	SD	12.25 ± 0.27^aD^	1.33 ± 0.04^aA^	9.78 ± 0.06^aA^

*Note:* Means ± standard deviation (*n* = 5). Results of the two‐way ANOVA‐based drying method. Different letters after mean values in columns represent significant differences at *p* < 0.05. Lowercase letters indicate statistical differences between sources, and capital letters describe the combined effect of both source and drying method. All results were reported on a dry basis (db).

Abbreviations: HAD, hot‐air drying; HPD, heat pump drying; SD, sun drying.

#### 3.1.2. The Impact of Drying Methods on the DF Composition of Guava

Regarding the results of guava, it was observed that SDF content varied remarkably across the different drying methods. The highest SDF content was in the HAD guava sample (8.12%), followed by the HPD (7.18%) and SD guava samples (5.54%), respectively. This mainly emphasizes the idea that SDFs, like pectin in the guava fruit, are broken down by the controlled and increased heat of the hot‐air oven, improving their extraction ability. This supports the notion reported by Hlaing [39] and Setyajati et al. [40], which signifies that increasing drying temperature enhances pectin solubilization in fruit matrices such as guava, apple pomace, and peels, and tropical fruits including papaya, lime, lemon, and dragon fruit, compared to lower temperatures. Although the drying temperature applied by the HAD (60 ± 2°C) is slightly moderate, a similar trend of temperature‐dependent solubilization may partly explain the observed variation in SDF content. It reveals the involvement of elevated thermal conditions in enhancing the extractability. Yusof et al. [41] found that in their studies, guava peel contains higher levels of pectin, ranging between 16.4% and 37.1%. Interestingly, the IDF content was highest in the SD guava sample (38.65%), surpassing both HAD (31.11%) and HPD (30.73%) samples. Only the SD guava sample exhibited significantly higher IDF content (*p* < 0.000) than the other two methods. The gentle drying conditions under ambient temperatures during SD may further preserve the structural integrity of IDFs, because cellulose, hemicelluloses, and lignin are less susceptible to heat‐induced degradation [42–44]. In contrast, Verma et al. [16] obtained an IDF content of 42.04% by drying guava in the hot‐air oven at 55°C, exceeding the IDF contents observed in the SD guava in the present study. This reflects the dynamic shift in the DF content influenced by variations in drying temperatures. The TDF content of the guava samples mirrored the pattern observed for IDF contents, aligned with the drying methods. The SD guava sample had the highest TDF concentration (44.46%), outperforming both HAD (39.31%) and HPD (38.09%) samples. However, it was observed that the TDF content was significantly changed (*p* < 0.000), underscoring the impact of drying methods. This aligns with the findings of Siriwattananon and Maneerate [20], validating that SD effectively maintains the highest DF content in guava compared to HAD and freeze‐dried forms. Their study also demonstrated the same pattern between dried pumpkin and cabbage samples [26].

#### 3.1.3. The Impact of Drying Methods on the DF Composition of Bitter Gourd

The SDF, IDF, and TDF contents of bitter gourd varied considerably with the different drying methods. The findings revealed that HAD bitter gourd showed 10.95% of SDF content, surpassing the levels found in HPD (6.26%) and SD (5.62%) samples. This finding was consistent with the study by Sungsinchai et al. [26], which showed that the SDF content of orange pulp residue was increased by 60°C of HAD. This is further congruent with findings from previous research highlighting the effectiveness of HAD in increasing SDF availability [34, 45]. In other words, the drying temperature strongly impacts SDF formation because high temperatures can degrade glycosidic bonds to enable transfer of larger DF fractions into more soluble, smaller formations [46]. In contrast, SD emerged as the drying method preserving the highest IDF (51.04%) and TDF (56.65%) contents in bitter gourd, highlighting the capability of SD to retain fibrous components. This outcome supports the findings of Garti et al. [47], who emphasized that SD caused a substantially high amount of DF and most other nutrients in the SD *Hibiscus cannabinus* leaves. Examining the IDF further, the HPD sample came in second with an IDF content of 38.15%, while the lowest value was in the HAD (36.17%) method. Conversely, the HAD sample exhibited the second‐highest TDF value (47.42%). In contrast, the HPD sample showed the lowest TDF value (44.51%). The bitter gourd sample dried in the hot‐air oven at 60 ± 2°C exhibited the lowest IDF and moderate TDF contents, while achieving the highest SDF content, indicating an exchange between solubilization and DF integrity, as previously mentioned [46].

#### 3.1.4. Overall Influence of Drying Methods on DF Composition Across Plant Sources

According to the study, the SDF, IDF, and TDF contents in mung bean, guava, and bitter gourd were significantly dependent on both the plant source and the drying method (*p* < 0.001) (Table 2). The results were consistent with previous findings [20, 48]. HAD was more successful at retaining fiber components than HPD or SD, as seen by the higher SDF, IDF, and TDF contents it produced overall. HAD retained the greatest SDF in bitter gourd (10.95%), supporting previous reports that HAD preserves fiber and nutrient stability [49]. However, SD produced the highest IDF (51.04%) and TDF (56.65%) in bitter gourd and similarly enhanced guava fibers. The superior fiber retention in SD samples of bitter gourd and guava may be attributed to structural preservation and the potential formation of new IDF under lower temperatures and extended drying durations [49, 50].

### 3.2. Influences of Drying Methods on Proximate Composition

Table 3 demonstrates the influences of different drying methods, such as HAD, HPD, and SD, on the composition pertaining to the crude protein, crude fat, and ash contents of mung bean, guava, and bitter gourd.

#### 3.2.1. Crude Protein Content

The analysis relevant to mung bean revealed that the sample dried using the HPD exhibited the highest crude protein content (32.57%), followed by the SD (31.05%) and HAD (28.15%) samples, respectively. The remarkable amount of protein retained in the HPD sample was primarily attributed to its optimal drying condition (40 ± 2°C for 12–18 h), which effectively reduces nutrient loss associated with excessive drying temperatures or prolonged drying times [51]. As mung bean is a legume, it naturally contains protein content that serves as a valuable plant‐based protein source with a protein content ranging between 20.97% and 31.32% and is susceptible to processing conditions due to its protein‐rich structure [52, 53]. Therefore, crude protein preservation reaches its optimal levels through the HPD process implementation. The crude protein content of guava showed notably higher content in both HAD (5.36%) and SD (5.15%) samples compared to the HPD (4.19%) sample. These results are consistent with the findings of Siriwattananon and Maneerate [20], who reported that the SD and HAD methods preserved more crude protein when compared to other drying methods. Ujong et al. [37] also reported similar findings that HAD amber date flour contained the highest crude protein content at 10.50%. Moreover, the HAD method enabled the production of tomato powder with a remarkable amount of protein retention of 3.70% [38]. This suggests that the drying method does not significantly affect the crude protein content within some specific plant sources, which is in line with the findings from Natumanya et al. [54], who reported that the nutrients and bioactive compounds in the selected vegetables remained largely unaffected across HAD, SD, and indirect SD methods.

#### 3.2.2. Crude Fat Content

The analysis of variance relevant to the results for crude fat content across plant sources, namely, mung bean, guava, and bitter gourd, revealed no statistically significant differences (mung bean: *p* > 0.375, guava: *p* > 0.863, and bitter gourd: *p* > 0.920) between the drying methods employed for each plant source. This implies that the drying methods cause no major changes in the lipids present in the tested samples. However, considering the interaction between plant sauces, the crude fat content of the bitter gourd sample was markedly higher than both the dried mung bean and guava samples. This observation aligns with the findings of Verma et al. [55], highlighting that the crude fat content of guava powder dried using four drying methods remained statistically equivalent. The study of Umoh et al. [56] revealed no noticeable differences regarding crude fat content in their work on HAD, SD, and solar‐dried green plantain flour that ranged between 2.26% and 2.51%. In comparison to the samples that were oven‐dried, the crude fat level of the SD bitter gourd was observed to be marginally lower. SD may induce sunlight‐mediated oxidation, particularly of unsaturated fatty acids, resulting in a reduction of total fat content in some dried samples compared to oven‐dried samples [56].

#### 3.2.3. Ash Content

The ash content indicates the total mineral composition of food while measuring the remaining inorganic substance following organic material and moisture separation [37, 56]. The experimental data revealed that guava showed considerable changes in ash content after drying through different methods, whereas no variations were observed in mung beans and bitter gourd through similar processes. Interestingly, it was observed that the SD guava sample demonstrated a slightly higher ash content (3.72%) compared to the HPD (3.54%) and HAD (3.53%) samples, respectively. This finding aligns with the observations of Umoh et al. [56], who reported that SD plantain had a higher ash content (4.28%) than HAD (3.94%).

#### 3.2.4. Overall Influence of Drying Methods on Proximate Composition Across Plant Sources

The present study demonstrates that all drying methods and plant sources have substantial effects on crude protein, crude fat, and ash contents of mung bean, guava, and bitter gourd, with strong statistical significance for protein (*p* ≤ 0.001), fat (*p* = 0.975), and ash (*p* = 0.989) (Table 3). HPD retained the highest protein levels due to gentle heating and shorter exposure times that limit denaturation [48, 49]. SD also preserved protein effectively in guava and bitter gourd, aligning with reports that ambient drying minimizes structural damage [20, 50]. HPD yielded the greatest crude fat content in bitter gourd, with only slight differences among methods, suggesting minimal lipid oxidation. Similarly, ash content remained relatively stable across methods, supporting earlier findings that minerals are resistant to thermal degradation during drying [48, 49].

### 3.3. Influences of Drying Methods on Hydration and OHCs

Table 4 demonstrates the influences of different drying methods, such as HAD, HPD, and SD on the OHC, WHC, and SWC of mung bean, guava, and bitter gourd.

**TABLE 4:** Variations of the hydration and oil holding capacities of dried mung bean, guava, and bitter gourd from different drying methods.

Source	Drying method	WHC (g g^−1^)	OHC (g g^−1^)	SWC (mL g^−1^)
Mung bean (*Vigna radiata)*	HAD	2.46 ± 0.09^cH^	1.43 ± 0.06^cG^	9.08 ± 0.28^bCD^
	HPD	3.38 ± 0.08^aF^	2.97 ± 0.09^aB^	12.51 ± 0.52^aB^
	SD	2.62 ± 0.07^bH^	1.68 ± 0.06^bF^	9.75 ± 0.43^bC^

Guava (*Psidium guajava*)	HAD	4.57 ± 0.05^bC^	2.51 ± 0.10^bC^	6.17 ± 0.76^bE^
	HPD	6.47 ± 0.07^aA^	3.40 ± 0.06^aA^	7.93 ± 0.86^aD^
	SD	4.29 ± 0.04^cD^	2.62 ± 0.04^bC^	5.75 ± 0.132^bE^

Bitter gourd (*Momordica charantia*)	HAD	3.18 ± 0.19^cG^	2.21 ± 0.05^bD^	9.27 ± 0.51^bC^
	HPD	5.07 ± 0.05^aB^	3.11 ± 0.05^aB^	15.49 ± 0.69^aA^
	SD	3.80 ± 0.05^bD^	1.95 ± 0.08^cE^	9.69 ± 0.44^bC^

*Note:* Means ± standard deviation (*n* = 5). Results of the two‐way ANOVA‐based drying method. Different letters after mean values in columns represent significant differences at *p* < 0.05. Lowercase letters indicate statistical differences between sources, and capital letters describe the combined effect of both source and drying method. All results were reported on a dry basis (db).

Abbreviations: HAD, hot‐air drying; HPD, heat pump drying; OHC, oil‐holding capacity; SD, sun drying; SWC, swelling water capacity; WHC, water‐holding capacity.

#### 3.3.1. WHC

The statistically analyzed results of mung bean, guava, and bitter gourd (Table 4) exhibited quantifiable variations in WHC values across different drying methods. Similarly, guava also exhibited its highest WHC in the HPD guava sample (6.47 g g^−1^), followed by HAD (4.57 g g^−1^) and SD (4.29 g g^−1^) samples. The pattern of WHC levels in bitter gourd was found to be comparable to that of mung beans, with the HPD method displaying a greater value (5.07 g g^−1^) compared to the HAD and SD. Notably, out of the three plant sources, the HPD method showed the highest WHC, indicating that it is better at retaining water‐binding components in the dried matrix, such as phenolics, carotenoids, and vitamins, which contribute to the water‐binding properties of HPD plant sources [57]. In HPD samples, plant sources undergoing controlled low‐temperature treatment led to minimal cellular damage due to the sufficient temperature management, which is understandable when compared to other conventional drying methods. This was reflected in the findings of Garcia‐Amezquita et al. [45], who demonstrated great water‐binding capacity values of freeze‐dried orange mango and prickly pear peel that exceeded their HAD samples.

#### 3.3.2. OHC

The HPD exhibited superior efficiency among other drying methods for preserving hydrophobic compounds such as carotenoids, lipids, and waxes due to their exceptional OHC retention levels. This pattern is consistent with previous studies that demonstrated the significance of the low‐temperature controlled drying methods in effectively preserving lipid‐related properties [58, 59]. As previously highlighted, HPD mung bean exhibited the highest OHC value at 2.97 g g^−1^, followed by SD (1.68 g g^−1^), and HAD achieved the lowest OHC value at 1.43 g g^−1^. Following the same trend, guava and bitter gourd showed maximum OHC values under HPD, reaching 3.40 and 3.11 g g^−1^, respectively. These findings indicate that the controlled drying environments of HPD play a critical role in more effectively preserving nutrients and phytochemicals like oil‐binding components compared to HAD and SD [58]. In guava, the OHC values of dry samples from SD and HAD displayed equivalent statistical results. Conversely, the OHC reached its lowest point when SD bitter gourd yields an OHC value of 1.95 g g^−1^. This indicates an underlying reduction in oil retention under this drying condition.

#### 3.3.3. SWC

Table 4 shows the statistically analyzed data from mung bean, guava, and bitter gourd, revealing substantial SWC variations among different drying methods. The HPD plant sources achieved the highest SWCs of 12.51, 7.93, and 15.49 mL g^−1^, respectively, for mung bean, guava, and bitter gourd. Interestingly, there was no statistically significant difference (*p* > 0.05) between the HAD and SD methods in the SWC values of all three samples. The controlled drying environment and optimal humidity conditions provided by heat pump dryers appear to be associated with enhancing functional properties such as SWC [51]. This is further confirmed by Garcia‐Amezquita et al. [45], who found that the high temperature of the hot‐air dryer can lead to significant changes in SWC, as their findings showed that HAD mango and prickly pear peel concentrates had lower SWC values than freeze‐dried samples. In contrast, Lan et al. [60] reported in their study that SD notably enhanced the swelling power of the dried samples. This finding aligns with the results of the present study, where SWC values in mung bean and bitter gourd samples dried by the SD method were higher than their HAD samples.

#### 3.3.4. Overall Influence of Drying Methods on Hydration and OHCs Across Plant Sources

According to the study, the WHC, OHC, and SWC in mung bean, guava, and bitter gourd were significantly dependent on both the plant source and the drying method (*p* < 0.001) (Table 4). The highest WHC, OHC, and SWC were consistently achieved by HPD across all tested samples. For instance, bitter gourd reached a WHC of 5.07 g g^−1^ and a SWC of 15.49 mL g^−1^ under the HPD method, which greatly exceeded other methods of HAD and SD. Previous research studies have shown that the water and oil absorption were promoted by limited disruption to cell wall structure and better retention of functional groups during the HPD method [48, 50]. In contrast, HAD and SD plant sources showed lower hydration and oil‐holding capacities, likely due to heat exposure and extended drying times that reduced matrix integrity [20, 49]. Furthermore, it was demonstrated that the slow dehydration and longer thermal stress caused the consistent drop in functional properties in SD of guava and mung bean, which can reduce the preservation of soluble pectin and other hydrophilic compounds essential for hydration and oil holding.

### 3.4. Effect of Drying Methods on Color Variations

Table 5 exhibits the color properties of the fresh plant sources. And, Table 6 demonstrates the influences of different drying methods, namely, HAD, HPD, and SD, on the color attributes of mung bean, guava, and bitter gourd. These values imply how different drying methods affect the visual properties of the dried plant sources.

**TABLE 5:** Color properties of mung bean, guava, and bitter gourd before applying drying treatments.

Source	*L* ^∗^ (lightness)	*a* ^∗^ (red green)	*b* ^∗^ (yellow blue)
Mung bean (*Vigna radiata*)	36.34 ± 2.8^a^	−8.96 ± 0.97^a^	12.60 ± 1.68^a^
Guava (*Psidium guajava*)	49.74 ± 4.68^b^	−9.47 ± 1.57^b^	16.07 ± 3.08^b^
Bitter gourd (*Momordica charantia*)	59.28 ± 2.81^c^	−4.61 ± 2.40^b^	24.42 ± 1.95^b^

*Note:* Means ± standard deviation (*n* = 5). Results of the one‐way ANOVA‐based drying method. Different letters after mean values in columns represent significant differences at *p* < 0.05. Lowercase letters indicate statistical differences between sources.

**TABLE 6:** Variations of the color properties of dried mung bean, guava, and bitter gourd from different drying methods.

Source	Drying method	*L* ^∗^ (lightness)	*a* ^∗^ (red green)	*b* ^∗^ (yellow blue)	Total color change (ΔE)	Browning index (BI)
Mung bean (*Vigna radiata*)	HAD	74.96 ± 0.52^cC^	−3.47 ± 0.08^aH^	17.02 ± 0.38^bD^	17.82 ± 1.85^aD^	21.59 ± 0.51^aA^
	HPD	80.16 ± 0.78^aA^	−4.49 ± 0.11^cF^	17.49 ± 0.55^abD^	22.23 ± 1.99^bBCD^	20.05 ± 0.93^bA^
	SD	78.55 ± 0.34^bB^	−4.15 ± 0.06^bG^	17.83 ± 0.26^aD^	20.59 ± 2.09^abCD^	21.04 ± 0.38^abA^

Guava (*Psidium guajava*)	HAD	39.66 ± 0.47^bH^	5.64 ± 0.05^aA^	13.47 ± 0.38^bE^	18.74 ± 4.19^aD^	51.24 ± 0.89^bA^
	HPD	52.80 ± 0.25^aG^	5.19 ± 0.12^bB^	24.28 ± 0.65^aA^	17.29 ± 1.14^aD^	67.11 ± 1.89^aA^
	SD	53.30 ± 0.74^aG^	5.48 ± 0.28^abA^	23.99 ± 0.75^aA^	18.04 ± 1.26^aD^	65.85 ± 1.71^aA^

Bitter gourd (*Momordica charantia*)	HAD	56.65 ± 0.50^cF^	1.23 ± 0.14^aC^	20.95 ± 0.21^aB^	24.30 ± 2.24^bBC^	46.55 ± 0.58^aA^
	HPD	61.41 ± 0.33^bE^	−1.63 ± 0.05^cE^	18.81 ± 0.29^bC^	27.04 ± 2.50^bAB^	33.79 ± 0.75^cA^
	SD	65.19 ± 1.04^aD^	−0.11 ± 0.16^bD^	20.84 ± 0.27^aB^	30.99 ± 2.66^aA^	37.51 ± 0.65^bA^

*Note:* Means ± standard deviation (*n* = 5). Results of the two‐way ANOVA‐based drying method. Different letters after mean values in columns represent significant differences at *p* < 0.05. Lowercase letters indicate statistical differences between sources, and capital letters describe the combined effect of both source and drying method.

Abbreviations: HAD, hot‐air drying; HPD, heat pump drying; SD, sun drying.

The HPD mung bean sample exhibited the highest *L* ^∗^ value (80.16), the brightest appearance, and the most negative *a* ^∗^ value (−4.49), reflecting the most pronounced green hue among the SD and HAD samples. Interestingly, the *b* ^∗^ values of the dried mung bean samples were substantially consistent with a marginally higher value in the SD sample. The total color change (ΔE) ranged from 17.82 to 22.23 in mung bean, with the highest ΔE observed in the HPD sample. Notably, despite this high ΔE, HPD exhibited the lowest BI (20.05), among all treated samples. In contrast, the HAD and SD methods presented marginally higher BI values. In guava, both HPD and SD samples exhibited drastically higher *L* ^∗^ values. However, these resulted in the highest BI values despite yielding moderate ΔE. This suggests that HPD and SD processes significantly enhanced yellowness and browning effects in guava. In contrast, the HAD guava demonstrated a redder hue with the most positive *a* ^∗^ value (5.64). The highest *b* ^∗^ value in the dried guava samples was observed in the HPD sample (24.28), followed closely by the SD sample (23.99), emphasizing the effectiveness of preserving yellow tone. In contrast, the HAD guava indicated potential degradation of yellow pigments. According to the analyzed results, the SD bitter gourd sample displayed the highest *L* ^∗^ value (65.19), while the HAD sample had the lowest *L* ^∗^ value and was the darkest of the dried bitter gourd samples. The SD bitter gourd produced the highest ΔE (30.99), while the HAD sample recorded the highest BI (46.55), indicating that this method led to more intense browning of the bitter gourd. In contrast, the HPD resulted in the significantly lowest BI of 33.79 in the dried bitter gourd samples (*p* < 0.009). The HPD sample exhibited the most negative *a* ^∗^ value (−1.63), indicating the greenest hue across the dried bitter gourd samples. Ozsan Kilic et al. [61] explain different food attributes, including product color change as a result of drying processes. The findings of this study also demonstrated notable variations in color characteristics of the dried plant sources subjected to various drying methods, highlighting the superiority of HPD in maintaining the most natural colors in all dried samples compared to other drying methods. According to the research outcomes, temperature and humidity management throughout the drying process are critical factors in preserving product color. Similarly, the findings of Loemba et al. [62] and Mishra et al. [40] corroborate these observations, that HPD works excellently as a drying method to safeguard the natural colors of various agricultural products, including cereals, fruits, and vegetables. High‐temperature drying can affect the color characteristics of the dried products. Particularly, changes in *L* ^∗^, *a* ^∗^, and *b* ^∗^ values may be partly a result of reactions such as caramelization and the Maillard reaction that occur during drying with elevated temperatures [63, 64].

#### 3.4.1. Overall Influence of Drying Methods on Color Variations Across Plant Sources

The present study showed that the color properties (*L* ^∗^, *a* ^∗^, *b* ^∗^) were significantly affected by the drying method and plant source (*p* < 0.001) (Table 6). In general, the higher values of lightness (*L* ^∗^) were achieved by HPD across all samples, such as 80.16 and 61.41 in mung bean and bitter gourd, respectively. According to the previous studies, they were suggested that the increase in *L* ^∗^ resulted in rapid moisture removal and reduced rates of browning reaction, which helps to safeguard the original color and the fresh appearance of the plant sources [48, 50]. Guava showed the highest *a* ^∗^ and *b* ^∗^ values, particularly under HAD and HPD, indicating partial pigment conversion and better retention of yellow hues. Overall, HPD provided superior color stability and reduced darkening compared to HAD and SD, supporting its suitability for preserving visual quality in dried plant materials.

## 4. Conclusion

This study successfully quantifies the SDF, IDF, and TDF content, proximate composition, functional properties, and color attributes of specially grown mung bean (*Vigna radiata*), guava (*Psidium guajava*), and bitter gourd (*Momordica charantia*) samples under the typical environmental conditions, collected from Sri Lanka. Different drying approaches, namely HAD, HPD, and SD, significantly influence their DF composition and physicochemical properties, along with the functional properties. In general, the HAD outperformed the other drying methods in increasing the amount of SDF in all three plant sources. On the other hand, IDF and TDF were best preserved by SD, particularly in guava and bitter gourd. This implies that the gentle drying conditions of SD maintain the structural integrity of fibrous components. These findings are vital for selecting the optimal drying method to maximize DF retention in plant‐based food sources, which is essential for enhancing DF quality in functional food products. The HPD optimized protein retention, and the crude protein in bitter gourd demonstrated resistance to all drying methods. Crude fat and ash content remained largely unaffected, reinforcing the stability of lipids under varying drying conditions. Functional properties were also profoundly influenced, with HPD exhibiting the highest WHC, OHC, and SWC, favoring fiber porosity and hydration. The HPD resulted in superior color retention, indicating minimum ΔE values when compared to other drying methods, while the HAD samples appeared darker, having comparatively higher BI values than other drying methods. Ultimately, the selection of an appropriate drying method is crucial and should align with the desired nutritional, functional, and visual qualities of the final product. Among the studied methods, HAD emerged as the most efficient for boosting SDF content across all three plant sources analyzed. In contrast, SD excelled in enhancing IDF and TDFs, particularly noted in guava and bitter gourd. Moreover, HPD was found to be the most effective in preserving functional properties, such as WHC, OHC, and SWC, while also maintaining the natural coloration of the dried powders. These insights facilitate the development of food processing techniques for creating functional, fiber‐rich food products with superior hydration capacity, enhanced lipid interactions, and improved esthetic appeal.

## Funding

This research was funded by the Research Council, University of Sri Jayewardenepura, Sri Lanka, Grant Number: ASP/01/RE/TEC/2022/72.

## Conflicts of Interest

The authors declare no conflicts of interest.

## Data Availability

The data supporting this study’s findings can be accessed through the corresponding author upon a reasonable request.
